# Inhibiting glycogen synthase kinase 3 suppresses TDP-43-mediated neurotoxicity in a caspase-dependent manner

**DOI:** 10.21203/rs.3.rs-6527592/v1

**Published:** 2025-05-29

**Authors:** Matthew Anthony White, Leon Crowley, Francesca Massenzio, Xingli Li, Michael Niblock, Michael Philip Coleman, Sami J Barmada, Jemeen Sreedharan

**Affiliations:** King’s College London; King’s College London; King’s College London; University of Michigan–Ann Arbor; King’s College London; University of Cambridge; University of Michigan–Ann Arbor; King’s College London

**Keywords:** TDP-43, GSK3 inhibition, ALS-FTD, Neurodegeneration, Caspase-dependent cleavage, Neurotoxicity attenuation, TDP-43 C-terminal fragments, Kinase inhibition

## Abstract

Amyotrophic lateral sclerosis (ALS) and frontotemporal dementia (FTD) are progressive and ultimately fatal diseases characterised by 43-kDa TAR DNA-binding protein (TDP-43) pathology. Current disease modifying drugs have modest effects and novel therapies are sorely needed. We previously showed that deletion of glycogen synthase kinase-3 (GSK3) suppresses TDP-43-mediated motor neuron degeneration in *Drosophila*. Here, we investigated the potential of GSK3 inhibition to ameliorate TDP-43-mediated toxicity in mammalian neurons. Expression of TDP-43 both activated GSK3 and promoted caspase mediated cleavage of TDP-43. Conversely, GSK3 inhibition reduced the abundance of full-length and cleaved TDP-43 in neurons expressing wild-type or disease-associated mutant TDP-43, ultimately ameliorating neurotoxicity. Our results suggest that TDP-43 turnover is promoted by GSK3 inhibition in a caspase-dependent manner, and that targeting GSK3 activity has therapeutic value.

## Introduction

Amyotrophic lateral sclerosis (ALS) and frontotemporal dementia (FTD) are progressive and fatal neurodegenerative diseases that exist on a clinicopathological spectrum (ALS-FTD)[1]. Clinically, ALS is characterised by motor dysfunction, while FTD leads to a decline in cognition affecting executive functions, behaviour and language capabilities. The available disease-modifying drugs have only a minor impact on survival and disease progression, and novel therapeutic agents are therefore urgently required.

Almost all ALS and half of FTD cases are characterised by cytoplasmic ubiquitinated inclusions positive for TAR DNA-binding protein 43 kDa (TDP-43)[2–4]. Disease-linked mutations in *TARDBP* (the gene encoding TDP-43) indicate a fundamental role for TDP-43 in ALS-FTD pathogenesis[5–8]. TDP-43 inclusions are also seen in Alzheimer’s disease[9, 10], Parkinson’s disease[11, 12], Huntington’s disease[13] and limbic-predominant age-related TDP-43 encephalopathy (LATE)[14]. These observations implicate aberrant homeostasis of TDP-43 in a broad range of neurodegenerative diseases.

Caspases[15–18] and calpains[19, 20] can cleave TDP-43 to generate 25, 35 and 42 kDa C-terminal fragments. The accumulation of these phosphorylated and aggregated C-terminal fragments is a hallmark of ALS-FTD[3, 21, 22]. Cleavage products of TDP-43 can be degraded by the proteasome and through autophagy[23–25]. However, whether aggregated and cleaved TDP-43 mediate disease or are non-toxic byproducts of physiological TDP-43 processing[26,27] is unclear.

Glycogen Synthase Kinase-3 (GSK3) is a highly conserved and ubiquitously expressed serine/threonine protein kinase with wide-ranging biological functions including glycogen metabolism, cell proliferation and apoptosis[28]. Mammalian GSK3 is encoded by two gene paralogues, *GSK3A* and *GSK3B*, which give rise to two protein isoforms GSK3α and GSK3β. Several lines of study link GSK3 biology to ALS-FTD pathogenesis. Firstly, expression of GSK3 is significantly increased in thoracic spinal cord tissue of patients with apparently sporadic ALS[29] and increased expression of GSK3 isoform β can be seen in frontal, hippocampal, cerebellar, cervical and lumbar tissue of patients with ALS or ALS with cognitive impairment[30]. Secondly, TDP-43 expression induces GSK3[31] whose activity modulates ER-mitochondrial associations regulated by vesicle-associated membrane protein-associated protein-B[32]. Thirdly, GSK3 is a modulator of TDP-43 cytosolic accumulation during cellular stress and its inhibition reduces the cytosolic accumulation of C-terminal TDP-43 fragments[33]. Finally, in an unbiased *in vivo* screen we previously showed that deletion of *shaggy*, the *Drosophila* orthologue of GSK3, significantly suppresses TDP-43-induced motor axon and neuromuscular junction degeneration[34]. Collectively, these data suggest that increased GSK3 plays a key role in neurodegeneration associated with TDP-43. Here, we confirm that GSK3 inhibition mitigates TDP-43-linked neurodegeneration in mammalian neurons and explore the biochemical and cellular mechanisms underlying this protective effect.

## Materials and Methods

### Mouse breeding and maintenance

This study was conducted on tissues from wild-type C57BI/6 J mice (*Mus musculus*) and rats (Rattus norvegicus) with breeding carried out in the UK and USA. All rodent work in the UK was conducted in accordance with the United Kingdom Animals (Scientific Procedures) Act (1986) and the United Kingdom Animals (Scientific Procedures) Act (1986) Amendment Regulations 2012. Experiments in the USA were approved by the Committee on the Use and Care of Animals (UCUCA) at the University of Michigan and performed in accordance with UCUCA guidelines. Mice were housed in cages of up to five animals under a 12 h light/dark cycle and rats were housed singly in chambers equipped with environmental enrichment.

### Plasmid constructs and small molecule compounds

The GFP-tagged TDP-43 expression constructs TDP-43^WT^ and TDP-43^Q331K^ were adapted from previously generated plasmids[6] by amplification of the TDP-43 open reading frame and ligation into the pEGFP-N1 vector. TDP-43^N-Del^ was generated by deletion of the first 81 amino acids from the N-terminus of TDP-43^WT^ using the QuickChange Site-Directed Mutagenesis Kit (Agilent).

The GSK3 inhibitor CHIR99021, CAS: 252917–06-9 was obtained from Abcam (ab120890) and a 100μM stock in DMSO stored at −20°C. AZD 1080, CAS:612487–72-6 was kindly provided by Dr Richard Mead, reconstituted in DMSO and stored at −80°C until use. The cell-permeable, irreversible caspase inhibitor Q-VD-OPh and broad-spectrum protein kinase inhibitor staurosporine were both reconstituted in DMSO and stored at −20°C.

### SH-SY5Y cell line culture

SH-SY5Y cells were maintained in DMEM/F-12, supplemented with GlutaMAX^™^ (Gibco, Thermo Fisher Scientific), 10% fetal bovine serum (FBS) (Gibco, Thermo Fisher Scientific), 1% Penicillin-Streptomycin (10,000U/ml, Thermo Fisher Scientific) and maintained at 37°C in a humidified 5% CO_2_ incubator.

### SH-SY5Y cell transfection and treatment

For western blots, cells were passaged, plated and allowed to recover for 24 h. Cells were transiently transfected with plasmid constructs expressing GFP or GFP tagged TDP-43^N-Del^, TDP-43^Q331K^ with TurboFect^™^ Transfection Reagent (Thermo Fisher Scientific) according to the manufacturer’s protocol. For cells treated with the small molecule GSK3 inhibitors, CHIR99021 or AZD1080, and the pan caspase inhibitor, Q-VD-OPh, compounds were administered at the time of transfection. The pro-apoptotic caspase activator staurosporine was administered 3 h before sample collection. After 24 h cells were lysed in RIPA buffer containing 10μg/ml protease and phosphatase inhibitor cocktail (Merck). Lysates were cleared by centrifugation and stored at −20°C until use.

For fluorescence imaging, cells were passaged and plated at a density of 1.5 × 10^4^ cells/well in CellCarrier-96 Ultra Microplates (Perkin Elmer) previously coated with Poly-DL-ornithine hydrobromide 100 mg (0.5mg/ml; Sigma). After 24h in culture, cells were transiently transfected in the same manner as for western blot experiments.

### Primary rat cortical neuron cell culture and transfection

Cortices from embryonic day (E)19–20 Long-Evans rat embryos were dissected and disassociated, and primary neurons were plated at a density of 6×10^5^ cells /ml in 96-well plates, as described previously[35]. At in vitro day (DIV) 4, neurons were transfected with 100ng EGFP to mark cell bodies and 50–100ng of GFP-tagged TDP-43 constructs using Lipofectamine 2000 (Invitrogen 52887), as previously described[36, 37, 38]. Following transfection, cells were placed in Neurobasal Complete Media (Neurobasal (Gibco 21103–049), 1x B27, 1x Glutamax, 100 units/mL Pen Strep (Gibco 15140–122)) and incubated at 37°C in 5% CO_2_. For compound treatments, neuronal media was supplemented at the time of transfection with either vehicle control or the GSK3 inhibitor CHIR99021 at concentrations ranging from 0.1μM to 10μM.

### Longitudinal fluorescence microscopy and automated image analysis

Neurons were imaged as described previously[39, 40] using a Nikon Eclipse Ti inverted microscope with PerfectFocus3a 20X objective lens and an Andor Zyla4.2 (+) sCMOS camera. A Lambda XL Xenon lamp (Sutter) with 5 mm liquid light guide (Sutter) was used to illuminate samples, and custom scripts written in Beanshell for use in μManager controlled all stage movements, shutters, and filters. Custom ImageJ/Fiji macros and Python scripts were used to identify neurons and draw both cellular and nuclear regions of interest (ROIs) based upon size, morphology, and fluorescence intensity. Fluorescence intensity of labelled proteins was used to determine protein localisation or abundance. Custom Python scripts were used to track ROIs over time, and cell death marked a set of criteria that include rounding of the soma, loss of fluorescence and degeneration of neuritic processes[36]. For measurement of nuclear and cytoplasmic protein levels, we performed automated analysis as described[39, 41]. Briefly, Hoechst vital nuclear dye was applied immediately after transfection. Nuclear ROIs were established by automated segmentation of the DAPI channel, while cellular ROIs were identified via a similar process in the RFP channel (corresponding to mApple fluorescence). The intensity of TDP-43-GFP constructs was then measured within the nuclear and cellular ROIs of each neuron, and cytoplasmic levels calculated as the difference between the cellular and nuclear ROIs.

### Primary motor neuron culture and transfection

Primary motor neurons were isolated and cultured from embryonic day 13.5 mouse embryos as previously described[42, 43]. Briefly, lumbar spinal cords were dissected, digested with trypsin and dissociated to a single cell suspension. Primary motor neurons were isolated by density gradient centrifugation using 6% Optiprep (Sigma) and cultured on glass coverslips coated with 0.5 mg/ml poly-ornithine (Sigma) and 0.5 mg/ml laminin (Thermo Fisher Scientific). Neurons were maintained in Neurobasal/B27 medium supplemented with 2% horse serum (Sigma), and 10ng/ml each of BDNF, CNTF, and GDNF (Peprotech) with 50% media exchanges every 3 days. Primary motor neurons were transfected by magnetofection as described[42]. Motor neurons were transfected at 2 DIV using magnetic nanobeads (NeuroMag, Oz Biosciences). Culture media was exchanged 1 hour prior to transfection with Neurobasal/B27 medium without serum. Plasmid DNA was incubated with NeuroMag in minimal essential medium (MEM) for 15 minutes, and then added dropwise to the cultures. Cells were incubated on top of a magnetic plate (Oz Biosciences) for 15 minutes and after removal of the magnet, media exchanged for complete neuronal media after 1 hour.

### Motor neuron survival assay

To quantify primary motor neuron survival, neurons were co-transfected with TDP-43 expression constructs and the pGL4.50[luc2/CMV/Hygro] luciferase reporter (Promega). After 4 DIV, luciferase expression was quantified using the Bio-Glo^™^ Luciferase Assay System (Promega) and a PHERAstar FS plate reader. Luciferase expression was used as a proxy for the number of surviving neurons. Assay reproducibility was confirmed by manual counting of GFP-TDP-43 positive cells.

### iPSC derived forebrain neuron culture and transfection

Differentiation of human forebrain neurons used a KOLF2.1J iPSC line with stable integration of a doxycycline inducible Neurogenin-2 (NGN2) expression cassette into the CLYBL safe harbour locus on chromosome 13. Stem cells were maintained in mTeSR plus media (Stemcell Technologies), routinely passaged using versene, and maintained on recombinant vitronectin coated plates (Thermo Fisher Scientific). To differentiate neurons, stem cells were single-cell dissociated using accutase and replated onto Geltrex coated dishes (Thermo Fisher Scientific) in stem cell media for 24 h with addition of a ROCK inhibitor (Merck). On day 1,2 and 3 post plating, media was exchanged for neuronal induction media consisting of DMEM-F-12/HEPES, 1x N2, 1x Glutamax, 1x Non-essential amino acids (Thermo Fisher Scientific) with the addition of 2mg/ml doxycycline (Merck) to induce expression of *NGN2*. After induction, neurons were dissociated with accutase treatment before replating into assay plates coated with a combination of Geltrex and laminin (Thermo Fisher Scientific). Neurons were maintained in neuronal maturation media consisting of Neurobasal Plus with addition of 1x Glutamax, 10ng/ml recombinant NT-3 (PeproTech) and 10ng/ml recombinant BDNF (PeproTech) with media exchanged twice weekly (50%).

Neurons were transfected using magnetofection using the same protocol as for primary mouse motor neurons above, with rodent neuron media replaced with human forebrain specific media.

### Statistical analyses

Statistical analyses were conducted using Prism 8.4.3 (GraphPad). For comparisons between genotypes or experimental groups, multiple *t*-tests or one-way ANOVA were used when comparing two or three groups, respectively. For comparison of means split on two independent variables, two-way ANOVA was used. Multiple comparisons were corrected using the Holm–Sidak test. For primary rat neuron survival analysis, the open-source R survival package was used to determine hazard ratios describing the relative survival between conditions through Cox proportional hazards analysis[36]. The statistical tests used and appropriate sample sizes are provided in the relevant figure legends. All statistical comparisons are based on biological replicates unless stated otherwise.

## Results

### TDP-43 undergoes N-terminally mediated caspase cleavage

**Figure 1:**
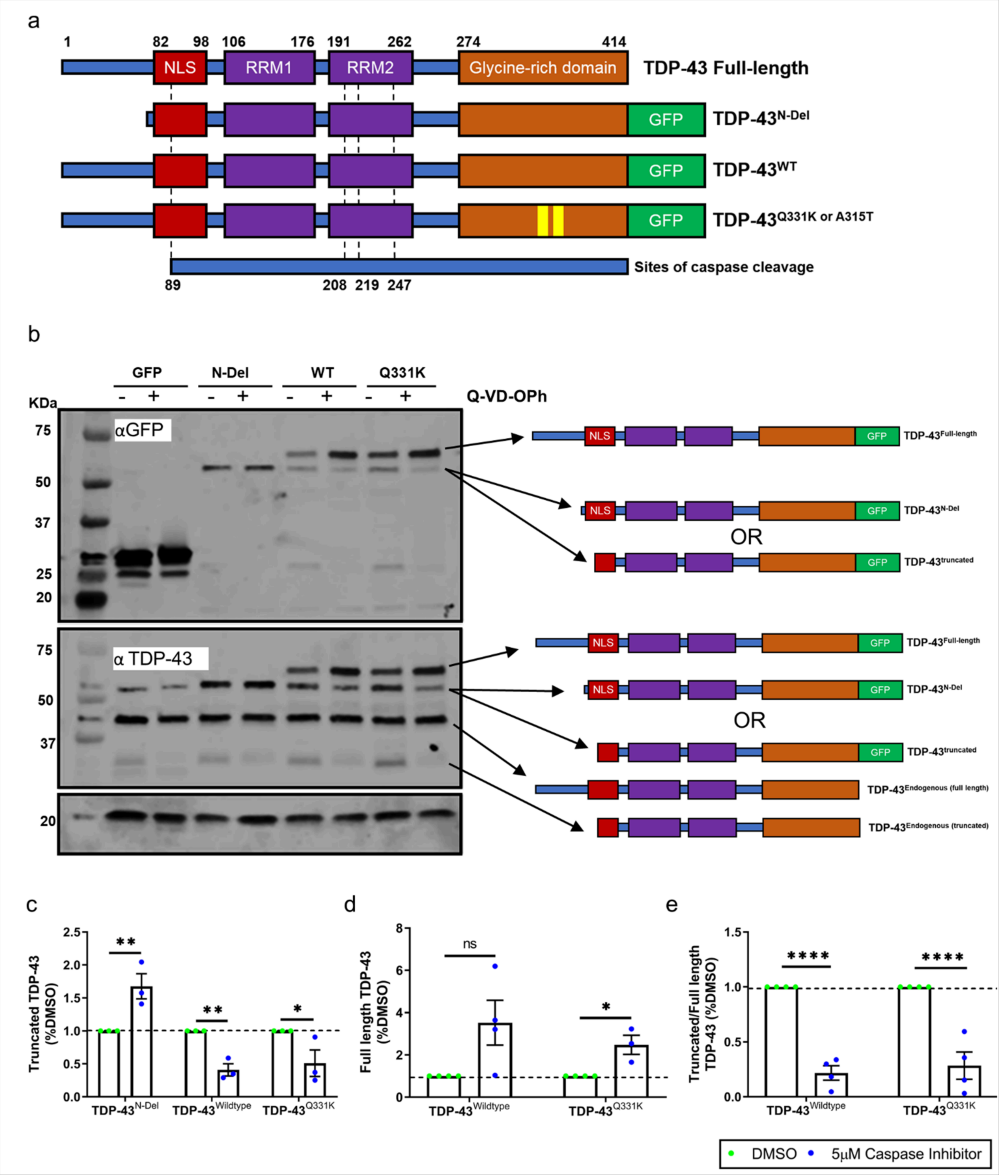
TDP-43 undergoes caspase-mediated cleavage **a.** Schematic of TDP-43 expression constructs used in this study. Dotted lines denote sites of caspase mediated cleavage of TDP-43. **b.** Representative immunoblot of SH-SY5Y cells transfected with expression constructs for GFP and GFP tagged: TDP-43^N-Del^, TDP-43^WT^ or TDP-43^Q331K^ for 24 hours with and without treatment with the pan caspase inhibitor Q-VD-OPh. **c-e**. Immunoblot band intensity quantifications showing the abundance of truncated, full length and the ratio truncated:full length TDP-43 in the absence of caspase activity (DMSO vs. Q-VD-OPh). **c.** Truncated TDP-43, pairwise comparisons: TDP-43^N-Del^: **P = 0.001679; TDP-43^WT^: **P = 0.004268; TDP-43^Q331K^: *P = 0.013091. **d.** Full length TDP-43, pairwise comparisons: TDP-43^WT^: ns *P* = 0.054872; TDP-43^Q331K^: *P = 0.011711. **e.** Ratio truncated:full length TDP-43. For **(c-e)** (n = 3–4 biological replicates per condition); ****P < 0.0001; multiple t test with Holm-Sidak correction for multiple comparisons. Error bars denote mean ± s.e.m.

To explore the mechanistic links between TDP-43 and GSK3 we began by expressing wild-type (TDP-43^WT^) and ALS-linked mutant (TDP-43^Q331K^) TDP-43 isoforms in human SH-SY5Y neuroblastoma cells. To control for nonspecific effects of transgene overexpression, we also transfected cells with an N-terminally truncated form of TDP-43 (TDP-43^N-Del^) (Fig. 1a). TDP-43^N-Del^ has several attractive features as a negative control as it lacks the region essential for dimerization and self-oligomerisation, which are critical steps necessary for many of the physiological functions of TDP-43 including nucleic acid binding [44]. N-terminal multimerization is also linked with the subcellular distribution of TDP-43 and its aggregation propensity[45–48]. To enable detection and comparative analysis across assays, all TDP-43 isoforms were C-terminally tagged with GFP (Fig. 1a).

We immunoblotted the transfected cell lysates for exogenous TDP-43 using an antibody recognising the GFP tag. Interestingly, cells expressing TDP-43^WT^ or TDP-43^Q331K^ demonstrated two prominent bands corresponding to full-length GFP-tagged TDP-43 and a smaller ~ 55kDa band, which was comparable in molecular weight to GFP-tagged TDP-43^N-Del^ (Fig. 1b). As the TDP-43^N-Del^ construct was deliberately truncated near to a caspase cleavage site (Fig. 1a), we hypothesised that the ~ 55kDa band seen after expression of full-length TDP-43 isoforms was a product of caspase cleavage. Indeed, application of the pan-caspase inhibitor Q-VD-OPh significantly reduced the abundance of the ~ 55kDa fragment and increased the abundance of full-length TDP-43^WT^ and TDP-43^Q331K^ (Fig. 1b–e). Truncation of endogenous TDP-43 can also be detected when immunoblotting with an antibody that recognises the full-length endogenous protein (Fig. 1b).

**Figure 2:**
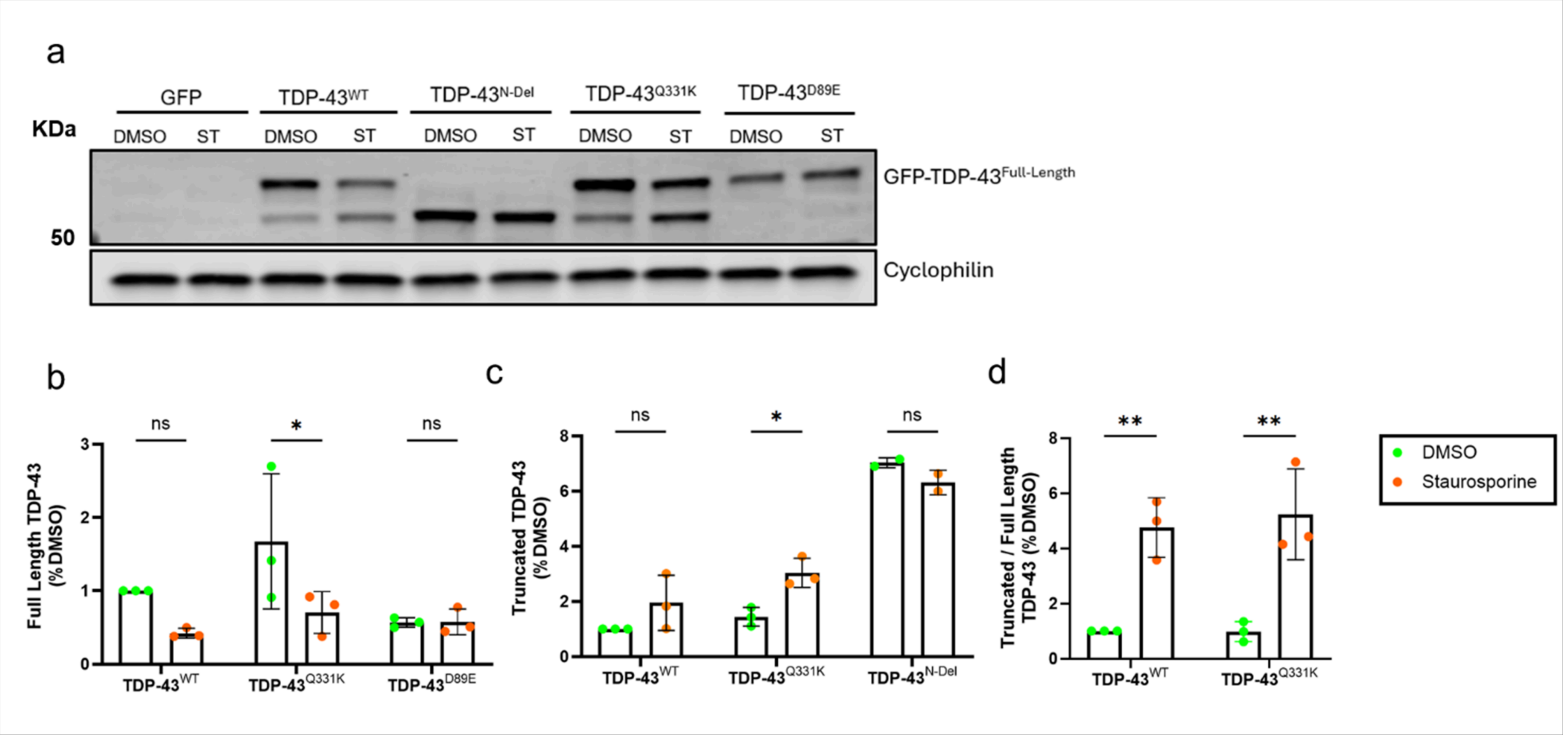
TDP-43 truncation is dependent on its N-terminal caspase recognition motif **a.** Representative immunoblot of SH-SY5Y cells transfected with expression constructs for GFP and GFP tagged: TDP-43^N-Del^, TDP-43^WT^, TDP-43^N-Del^, TDP-43^Q331K^ or TDP-43^D89E^ for 24 hours with and without treatment with the potent pro-apoptotic caspase activator staurosporine. **b-d.** Immunoblot band intensity quantifications showing the abundance of full length, truncated and the ratio truncated:full length TDP-43 (DMSO vs. staurosporine). **b.** Full length TDP-43 pairwise comparisons: TDP-43^WT^: ns *P* = 0.1973; TDP-43^Q331K^: *P = 0.0354; TDP-43^D89E^: ns *P* = 0.9795. **c.** Truncated TDP-43, pairwise comparisons: TDP-43^WT^: ns *P* = 0.1165; TDP-43^Q331K^: *P = 0.0156; TDP-43^N-Del^: ns *P* = 0.2216. **d.** Ratio truncated:full length TDP-43, pairwise comparisons: TDP-43^WT^: **P = 0.0018; TDP-43^Q331K^: **P = 0.0016. For (**b-d**) (n=3 biological replicates per condition); two-way ANOVA followed by Holm-Sidak *post-hoc* test for pairwise comparisons. Error bars denote mean ± s.e.m.

To further confirm a relationship between caspase cleavage and TDP-43 processing we treated cultures with staurosporine, a potent pro-apoptotic caspase activator. This resulted in an increase in the relative abundance of the ~ 55kDa TDP-43 band (Fig. 2a–d). This band was not evident in cells expressing the cleavage-resistant TDP-43^D89E^ mutant, a variant that removes the caspase recognition motif located in the N-terminal NLS of TDP-43 (Fig. 1a). Staurosporine treatment did not influence the abundance of full-length TDP-43^D89E^, nor truncated TDP-43^N-Del^. We conclude that overexpressed TDP-43 undergoes caspase-mediated cleavage to generate C-terminal TDP-43 fragments, and this event is mediated through the N-terminal caspase recognition motif located in the NLS.

### TDP-43 activates GSK3

**Figure 3:**
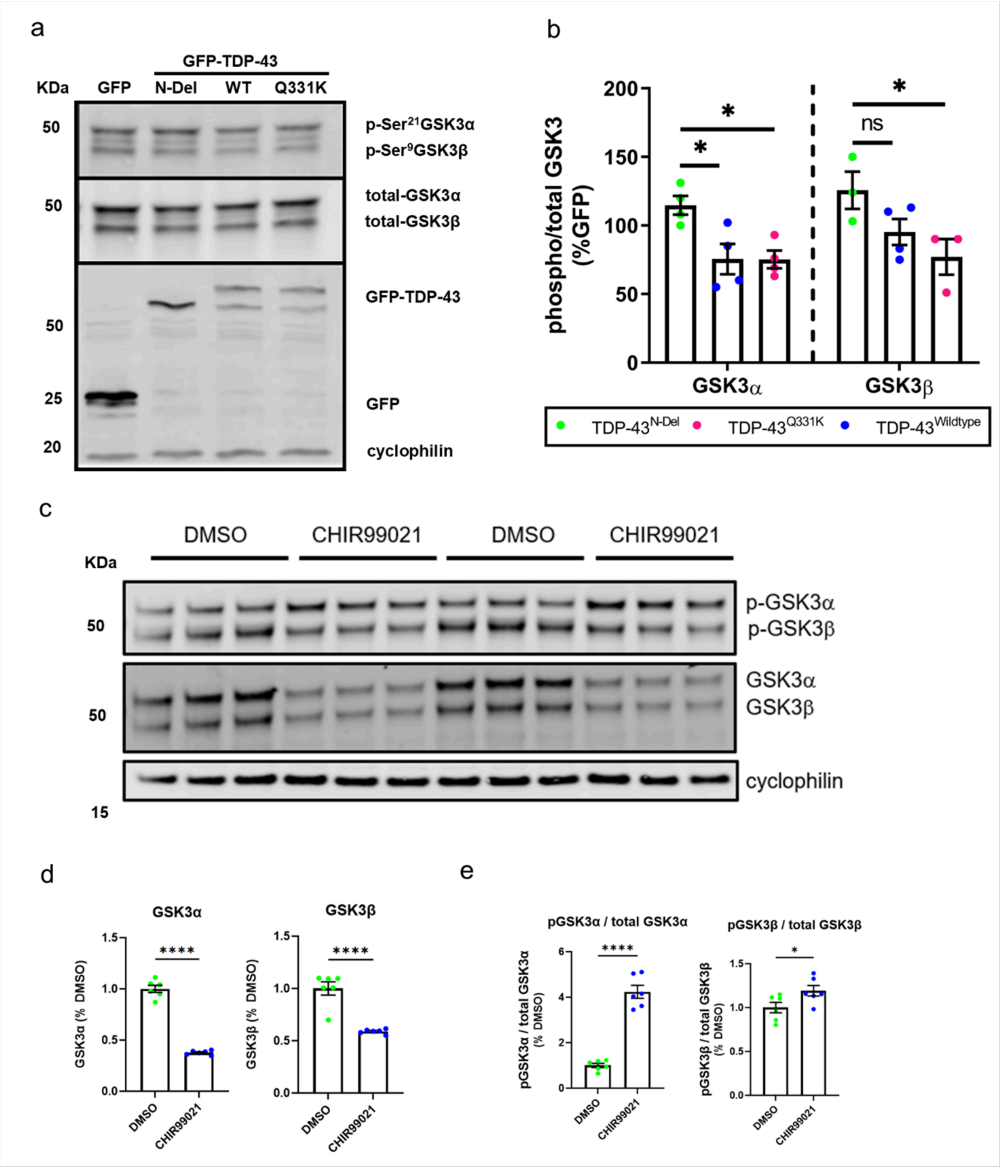
TDP-43 activates GSK-3 **a.** Representative immunoblot of SH-SY5Y cells transfected with expression constructs for GFP and GFP tagged: TDP-43^N-Del^, TDP-43^WT^ or TDP-43^Q331K^ for 24 hours and blotted for phospho-GSK-3α/β (Ser21/9) and total GSK-3α/β. **b.** Immunoblot band intensity quantification (*n* = 3–4 biological replicates per condition). The ratio between phospho-GSK3/total GSK-3 denotes GSK3 activation. ANOVA genotype *P* = 0.0012. Pairwise comparisons: GSK-3α, TDP-43^N-Del^ vs TDP-43^WT^: *P = 0.0186; TDP-43^N-Del^ vs TDP-43^Q331K^: *P = 0.0186; GSK-3β, TDP-43^N-Del^ vs TDP-43^WT^: ns *P* = 0.0515; TDP-43^N-Del^ vs TDP-43^Q331K^: *P = 0.0123. Two-way ANOVA followed by Holm-Sidak *post-hoc* test for pairwise comparisons. **c.** Representative immunoblot of SH-SY5Y cells with and without treatment with the small molecule GSK3 inhibitor CHIR99021 for 24 hours. **d-e.** Immunoblot band intensity quantifications showing the abundance of **d**. Total GSK-3a and GSK-3β. **e.** The ratio between phospho-GSK3a/total GSK3a and phospho-GSK3β/total GSK3β, pairwise comparisons: phospho-GSK3β/total GSK3β: *P = 0.0473, for **(d-e)** (n = 6 biological replicates per condition, DMSO vs. CHIR99021); ****p <0.0001; two-way ANOVA followed by Holm-Sidak *post-hoc* test for pairwise comparisons. Error bars denote mean ± s.e.m.

Immunoblotting of cell lysates for GSK3 demonstrated that TDP-43^Q331K^ increased the activation of both GSK3α and GSK3β, as evidenced by reduced phosphorylation of serine 21 and serine 9 respectively[49–51]. TDP-43^WT^ enhanced activation of GSK3α alone and TDP-43^N-Del^ had no significant effect on GSK3 activity (Fig. 3a–b). These observations are in keeping with previous studies demonstrating that TDP-43 activates GSK3[31]. Given this observation, we tested the commercially available small molecule GSK3 inhibitor CHIR99021 [52] to explore how it influences GSK3 function in our cell model and to facilitate downstream studies. Treatment of neuroblastoma cells with CHIR99021 for 24h resulted in a significant decrease in the abundance of GSK3α and β isoforms in addition to an increase in their phosphorylation, consistent with a reduction in GSK3 activity (Fig. 3c–e). CHIR99021 similarly reduced GSK3 activation in human induced pluripotent stem cell (iPSC) derived forebrain neurons, reducing the abundance of GSK3α and β isoforms in addition to significantly increasing the phosphorylation of GSK3α (Supplementary Fig. 1a-c).

### GSK3 inhibition preferentially reduces the abundance of truncated TDP-43

**Figure 4:**
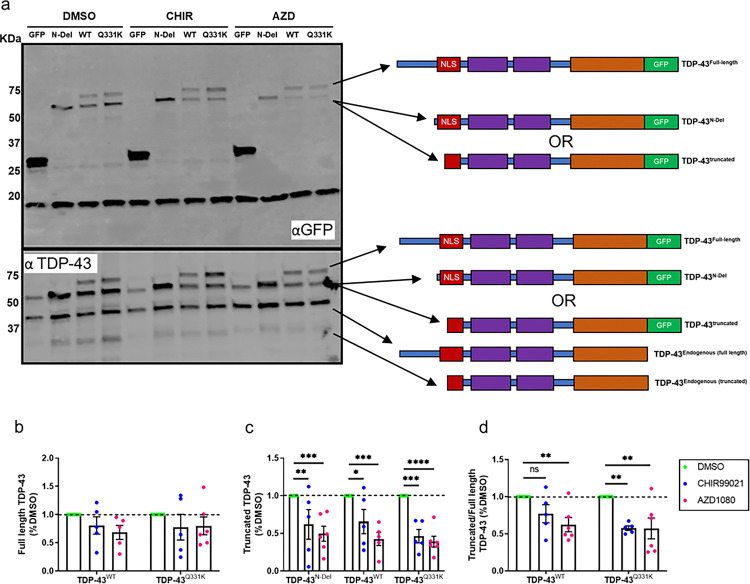
GSK-3 inhibition preferentially reduces the abundance of truncated TDP-43 **a.** Representative immunoblot of SH-SY5Y cells transfected with expression constructs for GFP and GFP tagged: TDP-43^N-Del^, TDP-43^WT^ or TDP-43^Q331K^ for 24 hours and treated with the GSK3 inhibitors CHIR99021 (CHIR) or AZD1080 (AZD). **b-d.** Immunoblot band intensity quantifications showing the abundance of full length, truncated and the ratio truncated:full length TDP-43 following treatment with CHIR or AZD. **b.** Full length TDP-43, ANOVA treatments ns *P* = 0.1216. **c.** Truncated TDP-43, pairwise comparisons: TDP-43^N-Del^, CHIR vs DMSO: ** *P* = 0.0077; AZD vs DMSO: *** *P* = 0.0007; TDP-43^WT^, CHIR vs DMSO: *P = 0.0156; AZD vs DMSO: ***P = 0.0003;TDP-43^Q331K^,CHIR vs DMSO: ***P = 0.0003;AZD vs DMSO: **** *P* < 0.0001. **d.** Ratio truncated:full length TDP-43, pairwise comparisons: TDP-43^WT^, CHIR vs DMSO: ns *P* = 0.0752; AZD vs DMSO: **P = 0.0071; TDP-43^Q331K^, CHIR vs DMSO: **P = 0.0024; AZD vs DMSO: **P = 0.0024. For **(b-d)** (*n* = 5–6 biological replicates per condition); two-way ANOVA followed by Holm-Sidak *post-hoc* test for pairwise comparisons. Error bars denote mean ± s.e.m.

To explore the link between GSK3 activity and TDP-43 fragmentation, SH-SY5Y neuroblastoma cells expressing TDP-43^N-Del^, TDP-43^WT^ or TDP-43^Q331K^ were treated with the GSK3 inhibitors CHIR99021 and AZD1080 (Fig. 4a). Subtle effects on the abundance of full length TDP-43^WT^ and TDP-43^Q331K^ were observed with GSK3 inhibition (Fig. 4b). More strikingly, however, GSK3 inhibition significantly reduced the abundance of cleaved products of both TDP-43^WT^ and TDP-43^Q331K^ (Fig. 4c,d). Beyond cleaved products, GSK3 inhibition also reduced the abundance of TDP-43^N-Del^ (Fig. 4c). To establish if GSK3 inhibition also reduces the abundance of endogenous TDP-43 we treated SH-SY5Y neuroblastoma cells (Supplementary Fig. 2a) and iPSC-derived forebrain neurons (Supplementary Fig. 2c) for 24h with CHIR99021. Treatment did not alter the cellular abundance of endogenous TDP-43 in either cell type suggesting that N-terminal cleavage and clearance is a response only to elevated expression of TDP-43 (Supplementary Fig. 2b,d). These results suggest that a GSK3-mediated mechanism alters the abundance of caspase-cleaved TDP-43 C-terminal fragments.

### GSK3 inhibition reduces the level of nuclear TDP-43 in a caspase-dependent manner

**Figure 5:**
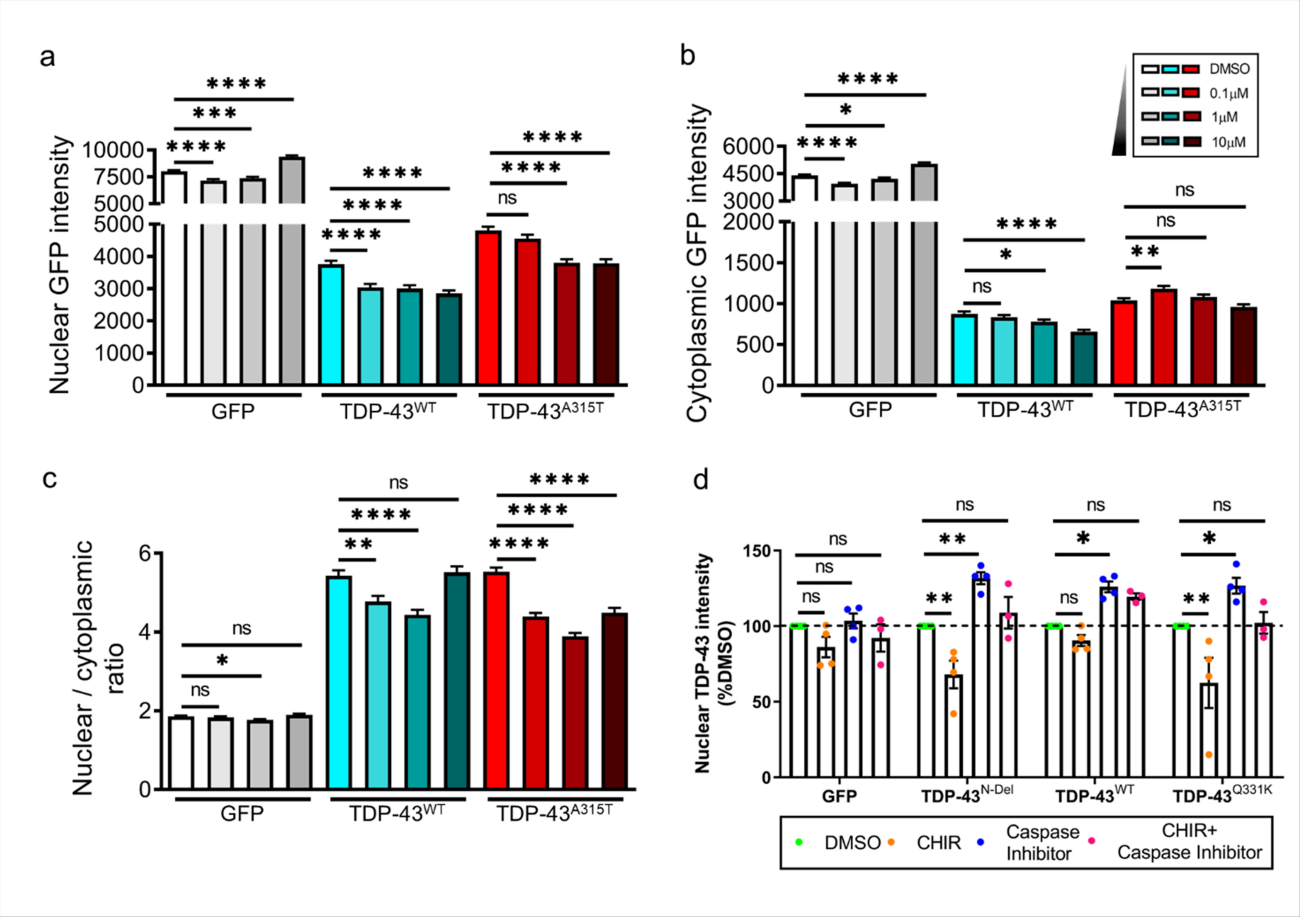
Inhibition of GSK3 reduces nuclear TDP-43 abundance in a caspase-dependant mechanism **a-c.** Subcellular distribution and abundance of GFP and GFP tagged TDP-43^WT^ and TDP-43^A315T^ following treatment with increasing doses of the GSK3 inhibitor CHIR99021 in cortical neurons. **a.** Nuclear TDP-43, pairwise comparisons: GFP, 1μm CHIR: ***P = 0.0003; TDP-43^A315T^,0.1μm CHIR: ns *P* = 0.1132. **b.** Cytoplasmic TDP-43, pairwise comparisons: GFP, 1μm CHIR: *P = 0.0256; TDP-43^WT^,0.1μm CHIR: ns *P* = 0.2529;1μm CHIR: *P* = 0.0292; TDP-43^A315T^, 0.1μm CHIR: *P = 0.0023; 1μm CHIR: ns *P* = 0.3181; 10μm CHIR: ns *P* = 0.1379. **c.** Ratio nuclear:cytoplasmic TDP-43, pairwise comparisons: GFP, 0.1μm CHIR: ns *P* = 0.4525; 1μm CHIR: *P = 0.0110; 10μm CHIR: ns *P* = 0.3138; TDP-43^WT^, 0.1μm CHIR: **P = 0.0018; 10μm CHIR: ns *P* = 0.6625. For **(a-c)** (*n* = 619–991 cells per condition from 3 technical replicate experiments); ****P < 0.0001; one-way ANOVA followed by Holm-Sidak *post-hoc* test for pairwise comparisons. **d.** Nuclear abundance of TDP-43 in SH-SY5Y cells transfected with expression constructs for GFP and GFP tagged: TDP-43^N-Del^, TDP-43^WT^ or TDP-43^Q331K^ for 24 hours. Cells are treated with the GSK3 inhibitor CHIR99021 and pan caspase inhibitor Q-VD-OPh. Pairwise comparisons: GFP, CHIR: ns *P* = 0.4423; Caspase inhibitor: ns *P* = 0.9809; CHIR + caspase inhibitor; ns *P* = 0.8586; TDP-43^N-Del^, CHIR: **P = 0.0062; Caspase inhibitor: **P = 0.0065; CHIR + caspase inhibitor; ns *P* = 0.8069; TDP-43^WT^, CHIR: ns *P* = 0.7312; Caspase inhibitor: *P = 0.0341; CHIR + caspase inhibitor; ns *P* = 0.2179; TDP-43^Q331K^, CHIR: **P = 0.0010; Caspase inhibitor: *P = 0.0278; CHIR + caspase inhibitor; ns *P* = 0.9962; (*n* = 3–4 biological replicates per condition); two-way ANOVA followed by Holm-Sidak *post-hoc* test for pairwise comparisons. Error bars denote mean ± s.e.m.

Cytoplasmic mislocalisation and nuclear depletion of TDP-43 are hallmarks of TDP-43 proteinopathies, at least at end-stage disease. To investigate the effects of GSK3 inhibition on TDP-43 localisation, primary rat cortical neurons were transfected with TDP-43 constructs C-terminally tagged with GFP (either TDP-43^WT^ or ALS-linked mutant TDP-43^A315T^). Neurons were subsequently treated with CHIR99021 in doses ranging from 0.1μM to 10μM. TDP-43-GFP intensity in the cytoplasm and nucleus was determined by automated high-content fluorescence microscopy, using a vital nuclear dye (Hoechst) as reference for the nuclear compartment, and a diffusely localised cellular marker (mApple) to outline the neuronal cytoplasm[39]. GSK3 inhibition by CHIR99021 significantly reduced the nuclear abundance of both TDP-43^WT^ and TDP-43^A315T^ in a dose-dependent manner (Fig. 5a). The effect on cytoplasmic TDP-43 was less pronounced, and was influenced by TDP-43 genotype: higher doses of the GSK3 inhibitor significantly reduced the abundance of TDP-43^WT^ but not TDP-43^A315T^ (Fig. 5b), thereby causing a reduction in the nuclear to cytoplasmic ratio of both TDP-43^WT^ and TDP-43^A31T^) (Fig. 5c). Given that the vast majority of TDP-43 is localised to the nucleus (Fig. 5a,b), inhibition of GSK3 effectively reduces the abundance of total cellular TDP-43^WT^ and TDP-43^A315T^ in a dose-dependent manner.

As both caspase inhibition and GSK3 inhibition reduce the abundance of N-terminally truncated TDP-43 (Fig. 1b,c and Fig. 4a,c), we hypothesised that caspase-cleavage and consequent disruption of the nuclear localisation sequence are key steps in the mechanism by which GSK3 regulates total cellular TDP-43. To test this hypothesis, we combined GSK3 inhibition with blockade of caspase activity. If N-terminal caspase cleavage occurs upstream of GSK3-mediated regulation of TDP-43, GSK3 inhibition should not alter TDP-43 expression in the absence of caspase activity. Neuroblastoma cells were transfected with constructs expressing GFP-tagged TDP-43^N-Del^, TDP-43^WT^ or TDP-43^Q331K^ and treated with either a GSK3 inhibitor, a pan-caspase inhibitor, or both in combination. We found that while inhibition of GSK3 reduced the nuclear abundance of TDP-43^N-Del^ and TDP-43^Q331K^, caspase inhibition had the opposite effect, causing an increase in nuclear TDP-43^N-Del^, TDP-43^WT^ and TDP-43^Q331K^ (Fig. 5.d). The ability of GSK3 inhibition to reduce the nuclear abundance of mutant TDP-43 was blocked when treated in combination with a caspase inhibitor (Fig. 5d). This indicates that GSK3 regulates the abundance of TDP-43 through an N-terminal caspase cleavage-dependent mechanism. Interestingly, the nuclear abundance of TDP-43^N-Del^ was also regulated in a similar manner to TDP-43^Q331K^. Although TDP-43^N-Del^ lacks much of the N-terminus, it retains the nuclear localisation sequence and caspase site. Thus, TDP-43^N-Del^ is still a target for caspase-mediated cleavage following GSK3 inhibition, and indeed CHIR99021 caused its levels to fall in a similar fashion to that of TDP-43^WT^ and TDP-43^Q331K^ (Fig. 4a,c).

### GSK3 inhibition ameliorates TDP-43 toxicity

**Figure 6:**
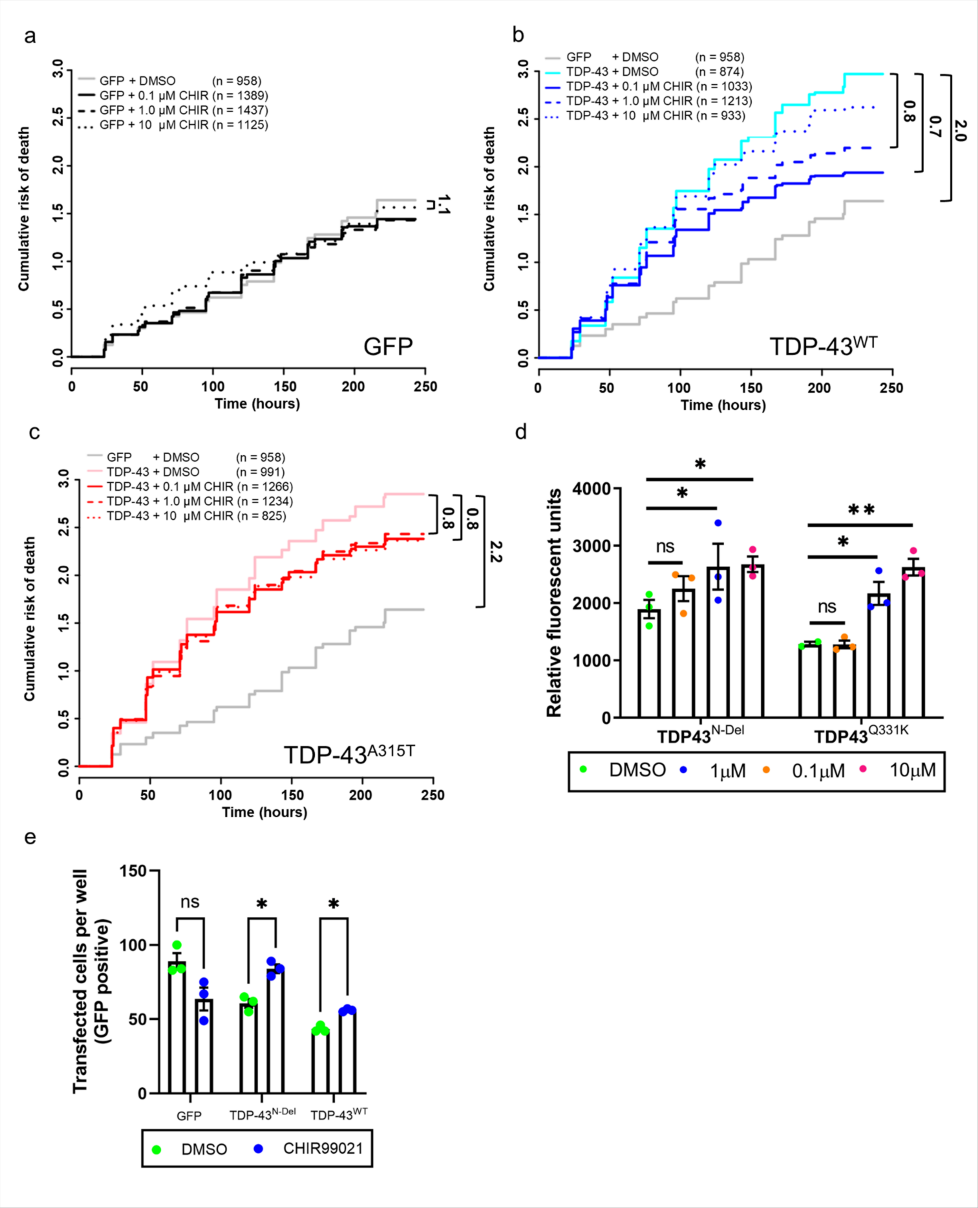
Small molecule inhibition of GSK3 ameliorates TDP-43 toxicity in rodent neurons and human iPSC-derived neurons **a-c.** Cumulative risk of death of primary rat cortical neurons expressing either GFP or GFP tagged TDP-43^WT^ and TDP-43^A315T^ treated with DMSO vehicle control or increasing doses of the GSK3 inhibitor CHIR99021 by longitudinal fluorescence microscopy. A hazard ratio above 1.0 indicates an increased risk of death while a value below 1.0 indicates the opposite. **a.** GFP expressing neurons, significant hazard ratios: GFP vs GFP + 0.1μM CHIR =1.1 **b.** TDP-43^WT^ expressing neurons, significant hazard ratios: GFP vs TDP-43^WT^ = 2.0; TDP-43^WT^ vs TDP-43^WT^ + 0.1μM CHIR = 0.7; TDP-43^WT^ vs TDP-43^WT^ +1.0μM CHIR = 0.8 **c.** TDP-43^A315T^ expressing neurons, significant hazard ratios: GFP vs TDP-43^A315T^ =2.2; TDP-43^A315T^ vs TDP-43^A315T^ + 0.1μM CHIR = 0.8; TDP-43^A315T^ vs TDP-43^A315T^ + 1.0μM CHIR = 0.8. The number of individual neurons tracked for risk of death analysis are displayed. d. Survival of primary mouse motor neurons expressing either TDP-43^N-Del^ or TDP-43^Q331K^ treated with increasing doses of CHIR. Pairwise comparisons: TDP-43^N-Del^, 0.1μM CHIR: ns *P* = 0.2381; 1.0μM CHIR: *P = 0.0492; 10μM CHIR: *P = 0.0492; Pairwise comparisons: TDP-43^Q331K^, 0.1μM CHIR: ns *P* = 0.9825; 1.0μM CHIR: *P = 0.0312; 10μM CHIR: **P = 0.0026; two-way ANOVA followed by Holm-Sidak *post-hoc* test for pairwise comparisons. **e.** Survival of iPSC-derived forebrain neurons expressing either GFP, TDP-43^N-Del^ or TDP-43^WT^ treated with CHIR. Pairwise comparisons (DMSO vs CHIR99021): GFP: ns *P* = 0.2381, TDP-43^N-Del^: ns *P* = 0.2381, TDP-43^WT^: ns *P* = 0.2381); multiple t test with Holm-Sidak correction for multiple comparisons. For **(d-e)** *n* = 3 biological replicates per condition. Error bars denote mean ± s.e.m.

We previously found that loss of GSK3 suppressed TDP-43-mediated neurodegeneration in *Drosophila melanogaster*[34]. To determine the therapeutic potential of targeting GSK3 in mammalian cells, we expressed GFP tagged TDP-43^WT^ or TDP-43^A315T^ in primary rat cortical neurons and treated them with CHIR99021. The viability of single transfected cells was tracked over time using robotic microscopy[36]. Expression of both TDP-43^WT^ and TDP-43^A315T^ significantly increased the cumulative risk of death relative to expression of GFP alone, increasing the hazard ratios by 2.0 and 2.2 respectively (Fig. 6a–c). We found that CHIR99021 reduced the risk of TDP-43-mediated neuronal death in a dose-dependent manner in those cells transfected with either TDP-43^WT^ or TDP-43^A315T^ (Fig. 6a–c). Similar results were obtained in mouse primary motor neurons expressing TDP-43^N-Del^ and TDP-43^Q331K^ (Fig. 6d) using an independent luciferase-based survival assay. As GSK3 inhibition can regulate the expression of TDP-43^N-Del^ in the same manner as full length TDP-43 (Fig. 4a,c and Fig. 5d), our results suggest that TDP-43^N-Del^ is processed in the same manner as full length variants. Inhibition of GSK3 improved motor neuron survival in cells expressing N-terminally truncated TDP-43 suggesting this truncated variant exerts a modest degree of toxicity to primary motor neurons despite its inability to dimerise and fully function. TDP-43^Q331K^ was significantly more toxic to primary mouse motor neurons than TDP-43^N-Del^, and addition of CHIR99021 significantly increased the survival of motor neurons expressing TDP-43^Q331K^.

To determine if there was a protective effect of GSK3 inhibition on human neurons we expressed GFP-tagged TDP-43^N-Del^ or TDP-43^WT^ in human iPSC-derived forebrain neurons and treated these with CHIR99021. GSK3 inhibition significantly improved the survival of neurons treated with both TDP-43^N-Del^ and TDP-43^WT^ameliorating TDP-43 toxicity in human neurons (Fig. 6e), confirming the neuroprotective effect of GSK3 inhibition across multiple model systems.

## Discussion

### Inhibition of GSK3 promotes neuronal survival and decreases the abundance of TDP-43 in a caspase-dependent manner

Here, we have shown that inhibition of GSK3 enhances the survival of both cortical and motor neurons expressing TDP-43^WT^ or the ALS-linked mutants TDP-43^Q331K^ and TDP-43^A315T^. In addition to its survival promoting effects, inhibition of GSK3 reduces the cellular level of caspase-cleaved TDP-43 C-terminal fragments. Caspase activity is a key requirement for both the reduction in TDP-43 abundance and survival promoting effect of GSK3 inhibitors. This suggests that GSK3 inhibitors act to enhance the turnover of TDP-43 through a caspase dependent mechanism. The result is a reduction in TDP-43 levels, which promotes neuronal survival, an observation that is in keeping with our previous finding in *Drosophila*[34]. The results we present here highlight an intriguing facet of TDP-43 cleavage, which is that the production of C-terminal fragments may have beneficial consequences. Rather than being a toxic process, cleavage of TDP-43 by caspases can cause a reduction in the abundance of full-length TDP-43, which promotes cellular survival.

### A positive feedback loop in the TDP-43-GSK3 axis could contribute to neurodegeneration

In support of our findings, a growing body of evidence indicates that inhibition of GSK3 is neuroprotective. GSK3 inhibition significantly delays disease onset and prolongs lifespan in the SOD1^G93A^ mouse model of ALS[53–55] and the GSK3 inhibitor kenpaullone prolongs survival of human iPSC-derived motor neurons harbouring the SOD1^L144F^ or TDP-43^M337V^ mutations[56]. Chronic inhibition of GSK3 by lithium is neuroprotective against kainate-induced excitotoxic motor neuron death in organotypic slice cultures[57]. Ghrelin, a circulating hormone produced by enteroendocrine cells, protects spinal motor neurons against glutamate-induced excitotoxicity in part through PI3K/Akt-mediated inactivation of GSK3β[58]. Inhibitors of GSK3 abrogate accumulation of C-terminal TDP-43 fragments in transfected cells[33], and protect motor neurons from neuroinflammation-induced degeneration[59]. Thus, GSK3 is an attractive target for therapeutic intervention in TDP-43 linked neurodegeneration.

While inhibition of GSK3 influences TDP-43 abundance, it is also notable that TDP-43 can activate GSK3[32]. Furthermore, the abundance of GSK3β is also increased in the frontal and temporal cortices of patients with ALS and concomitant cognitive impairment[30]. Expression of TDP-43 perturbs the ER-mitochondria interface by disrupting interaction between VAPB and PTPIP51 through GSK3β activation[32]. Thus, TDP-43 and GSK3 are fundamentally linked in a reciprocal manner, with mis-regulation of one impacting the function of the other. This intimate relationship raises the possibility that elevated TDP-43 could act in a positive feedback loop by activating GSK3 to negatively impact its own turnover. In such a situation, the abundance of TDP-43 would increase over time as its GSK3-mediated clearance is increasingly inhibited.

### TDP-43 abundance must be tightly controlled for cellular viability

TDP-43 binds a large proportion of the transcriptome and regulates several key steps of RNA metabolism[2, 60–64]. Minor alterations in TDP-43 abundance cause widespread transcriptomic changes that impact cellular function, so it is critical that mechanisms are in place to carefully regulate TDP-43 levels[65, 66]. Indeed, the level of TDP-43 is exquisitely controlled by a process of autoregulation, disruption of which is linked with ALS-FTD[65, 67–70]. Our results indicate that disruption of the caspase-dependent GSK3-TDP-43 axis is another route by which TDP-43 levels may rise with toxic consequences. At the endoplasmic reticulum, TDP-43 has been shown to be cleaved at amino acid 174 by membrane bound caspase-4 generating a 25 kDa C-terminal fragment[24, 71]. Subsequent activation of caspase-3/7 cleaves full length TDP-43 to produce a 35 kDa fragment. This sequential fragmentation reduces the abundance of TDP-43, and mitigates cytotoxicity caused by TDP-43 overexpression[24]. TDP-43 overexpression initiates caspase-4 cleavage of TDP-43 before the onset of detectable ER stress and represents a physiological mechanism to control its abundance, rather than a pathological mechanism triggered by ER stressors[25]. Thus, the fact that TDP-43 levels are tightly regulated at both RNA and protein levels emphasises the importance of TDP-43 homeostasis for cellular health.

### Misregulation of TDP-43 in neurodegenerative disease

The cleavage and aggregation of TDP-43 in the brains of ALS-FTD patients suggests that homeostatic mechanisms regulating TDP-43 processing have been overwhelmed in disease[3]. Misregulation of TDP-43 can arise in several contexts and contribute to pathological phenotypes. The ALS associated Q331K mutation perturbs TDP-43 autoregulation thereby increasing the abundance of TDP-43[65]. Patient-derived TDP-43^M337V^ neurons have increased TDP-43 expression[72] and spinal motor neurons of patients with apparently sporadic ALS have elevated *TARDBP* mRNA[73]. The untranslated regions (UTRs) of *TARDBP* contain regulatory elements responsible for transcript stability and control, and patients with ALS demonstrate an increase in the burden of rare genetic variants in these UTRs[74]. One of these variants (c.*2076G > A in two patients with ALS-FTD) was shown to result in a doubling of TARDBP mRNA[75]. Under the burden of excessive *TARDBP* transcription, processing of TDP-43 at the ER could potentially be overwhelmed and contribute to a toxic increase in the abundance of full length TDP-43.

### TDP-43 fragmentation is increased in disease

Caspase activation is a feature of several ER stressors[76] including aging[77], protein misfolding[78] and aggregation[79] and is increased in both the brains and spinal cords of patients with ALS [80]. Chemical induction of apoptosis, ER stress, chronic oxidative stress and D-sorbitol induced hyper osmotic pressure can all trigger the generation of 35 kDa TDP-43 fragments in a caspase dependent manner[25]. Human lymphoblastoid lines from patients harbouring *TARDBP* mutations show that mutant TDP-43 is predisposed to fragmentation [5, 15, 81] while overexpression of mutant TDP-43^A315T^ in HEK293 cells causes persistent accumulation of protease resistant TDP-43 fragments[82]. The most common genetic cause of ALS-FTD is a hexanucleotide expansion in *C9orf72*. Repeat-associated non-AUG (RAN) translation generates several toxic dipeptide repeats including a poly-GA protein from the repeat expansion. These poly-GA repeats induce expression of caspase-3, potentially linking *C9orf72* expansion and RAN-translation with TDP-43 proteolytic processing[83]. Levels of activated caspase-3 are also increased in spinal motor neurons of ALS patients with risk-modifying polyglutamine expansions derived from mutant *ATXN2*[84]. While the presence of C-terminal TDP-43 fragments are a clear pathological hallmark in ALS-FTD, overexpression of 35 kDa or 25 kDa TDP-43 fragments does not necessarily cause cell death[85] or neurodegeneration *in vivo*[86]. This further supports the hypothesis that caspase mediated cleavage of TDP-43 attenuates toxicity[85]. Further studies are warranted to explore how this mechanism could be targeted to alleviate TDP-43-mediated neurodegeneration.

## Conclusion

Multiple avenues of disease pathogenesis, influencing *TARDBP* transcript regulation, caspase cleavage of TDP-43 and GSK3 activity have the potential to disrupt cellular TDP-43 homeostasis. Exposure to environmental or pathological ER stressors, missense mutations that alter TDP-43 expression or a combination of several factors over time could lead to a gradual failure in the homeostatic maintenance of TDP-43, causing its accumulation. GSK3 inhibition reduces TDP-43 abundance in a cleavage-dependent manner, alleviating TDP-43-linked neurotoxicity. GSK3 inhibition therefore represents a target for therapy in ALS-FTD.

## Data Availability

The datasets used and/or analysed during the current study are available from the corresponding author on reasonable request.

## Abbreviations

(ALS-FTD): Amyotrophic lateral sclerosis-frontotemporal dementia
(TDP-43): 43-kDa TAR DNA-binding protein
(GSK3): Glycogen synthase kinase-3
(ALS): Amyotrophic lateral sclerosis
(FTD): Frontotemporal dementia
(LATE): Limbic-predominant age-related TDP-43 encephalopathy
(DIV): Day in vitro
(ROIs): Regions of interest
(NGN2): Neurogenin-2
(iPSC): Induced pluripotent stem cell
(UTRs): Untranslated regions
(RAN): Repeat-associated non-AUG
